# Integrating microRNA and mRNA expression profiles in response to radiation-induced injury in rat lung

**DOI:** 10.1186/1748-717X-9-111

**Published:** 2014-05-09

**Authors:** Ling Xie, Jundong Zhou, Shuyu Zhang, Qing Chen, Rensheng Lai, Weiqun Ding, ChuanJun Song, XingJun Meng, Jinchang Wu

**Affiliations:** 1 Department of Radio-oncology, Nanjing Medical University Affiliated Suzhou Hospital, Suzhou 215001, China; 2 School of Radiation Medicine and Public Health, Medical College of Soochow University, Suzhou 215123, China; 3 Department of Pathology, Affiliated Hospital of Nanjing University of Chinese Medicine, Nanjing 210029, China; 4 Department of Pathology, University of Oklahoma Health Science Center, Oklahoma City, OKC, USA

**Keywords:** Radiation, Rat lung, MicroRNAs, mRNA, Microarray

## Abstract

**Purpose:**

Exposure to radiation provokes cellular responses, which are likely regulated by gene expression networks. MicroRNAs are small non-coding RNAs, which regulate gene expression by promoting mRNA degradation or inhibiting protein translation. The expression patterns of both mRNA and miRNA during the radiation-induced lung injury (RILI) remain less characterized and the role of miRNAs in the regulation of this process has not been studied. The present study sought to evaluate miRNA and mRNA expression profiles in the rat lung after irradiation.

**Methods and materials:**

Male Wistar rats were subjected to single dose irradiation with 20 Gy using 6 MV x-rays to the right lung. (A dose rate of 5 Gy/min was applied). Rats were sacrificed at 3, 12 and 26 weeks after irradiation, and morphological changes in the lung were examined by haematoxylin and eosin. The miRNA and mRNA expression profiles were evaluated by microarrays and followed by quantitative RT-PCR analysis.

**Results:**

A cDNA microarray analysis found 2183 transcripts being up-regulated and 2917 transcripts down-regulated (P ≤ 0.05, ≥2.0 fold change) in the lung tissues after irradiation. Likewise, a miRNAs microarray analysis indicated 15 miRNA species being up-regulated and 8 down-regulated (P ≤ 0.05). Subsequent bioinformatics anal -yses of the differentially expressed mRNA and miRNAs revealed that alterations in mRNA expression following irradiation were negatively correlated with miRNAs expression.

**Conclusions:**

Our results provide evidence indicating that irradiation induces alterations of mRNA and miRNA expression in rat lung and that there is a negative correlation of mRNA and miRNA expression levels after irradiation. These findings significantly advance our understanding of the regulatory mechanisms underlying the pathophysiology of radiation-induced lung injury. In summary, RILI does not develop gradually in a linear process. In fact, different cell types interact via cytokines in a very complex network. Furthermore, this study suggests that microRNAs may serve an important role in the pathogenesis of RILI and that understanding their role in RILI may have a significant effect on patient management and diagnosis in the future.

## 

Radiation-induced lung injury (RILI) is a major dose-limiting toxicity in thoracic radiotherapy (RT), occurring in approximately 10-20% of patients who undergo radio -therapy to the thorax [1]. It is manifested with two distinct phases: an early phase characterized as radiation pneumonitis (acute, 1-6 months post-RT) and a late phase of the development of pulmonary fibrosis (>6 months post-RT) [2]. Clinical symptoms range from cough, fever, and shortness of breath to death from respiratory failure. Modern treatments, including image-guided radiotherapy, are still limited by its toxicity on normal tissues, thus impeding further gains with these technologies. In addition, most of the chemotherapeutic agents used in conjunction with radiotherapy increases the risk of damage to normal tissues. Thus, attenuating radiation-induced normal tissue damage is of critical importance in improving tumor control and the quality of patient life. The development of new therapies or strategies will, however, require an improved understanding of the basic molecular mechanisms that underlie the development of radiation-induced lung injury.

MicroRNAs (miRNAs) are non-coding small RNA molecules of 21 to 24 nucleotides, which regulate gene expression by targeting the 3′-untranslated regions of the RNA transcripts. Recent advances in the understanding of miRNA mechanisms of activity biology have demonstrated that miRNAs are involved in many biological activities such as stem cell differentiation, organ development, cell death, phase change of the cell cycle, and signal transduction [3]. The involvement of miRNAs in human diseases such as cancer has also been demonstrated. Of note is that miRNAs have recently been implicated in cellular responses to oxidative stress, DNA damage, oncogenic stress, and irradiation where genetic knockdown of specific miRNA renders the mutant animals incapable of coping with these stressors [4]. Of clinical importance, the expression of distinct miRNAs seem to be associated with the prognosis of the efficacy of therapeutic interventions, including radiotherapy [5]. In fact, miRNAs have been shown to modulate the radiosensitivity of lung cancer cells, breast cancer cells *in vitro* and Caenorhabditis elegans *in vivo*[6,7]. Moreover, normal cells show altered levels of miRNAs in response to ionizing radiation [8].

The data available on the histopathologic changes after RT of human lung are limited, because patients undergoing lung RT are unlikely to undergo diagnostic thoracotomy and autopsies are rarely performed. The existing data have mostly come from animal models. Both the acute response of the lung to radiation and the ultimate development of radiation pneumonitis and fibrosis have been well documented in small animal models (rat and mice). Combining respiratory function, chest CT, and pathological changes, RILI in these animal models were divided into early period (0-8weeks), middle period (8-12 weeks), stable period (12-16 weeks), and late period (16-26 weeks) [9,10]. In the present study, we observed a sequence of morphological changes in the lungs of rat from the early to the late stage following a single dose irradiation with 20 Gy to the right lung, based on a pilot study and previous reports [11].

Evidence has suggested a central role for inflammation in the initiation and establishment of radiation-induced pneumopathy [12]. We believe that the process of acute pneumonitis and radiation fibrosis are tightly linked with each other. Radiation -induced oxidative stress and free radical generation triggers inflammation and results in DNA damage in all of the lung cellular components, inducing mRNA transcription of a variety of genes involved in epithelial/connective tissue and vascular regeneration. At the cellular level, radiation pneumonitis is characterized by lymphocytic alveolitis. This phase of the direct induction of growth factor, adhesion molecule, and cytokine over-expression by ionizing radiation is probably the first event that triggers the subsequent cascade of radiation pneumopathy. The expression of inflammatory cytokines, chemokines, adhesion molecules and their receptors, as well as the resultant cellular interactions, appear to stimulate the subsequent expression of fibrotic cytokines, such as TGF-β, basic fibroblast growth factor (bFGF) and vascular endothelial growth factor (VEGF). These cytokines participate in the up-regulation of extracellular matrix deposition that progressively leads to lung fibrosis [13,14].

Although RILI has been extensively studied, the molecular mechanisms underlying the pathophysiological changes in the lung induced by radiation remain to be defined. Moreover, information is limited concerning changes in miRNAs expression within the lung during irradiation, and there has been no reports describing miRNA -mRNA interactions in RILI. Thus, the present study was aimed to integrate miRNA and mRNA expression profiles in the rat lung after irradiation using Agilent mRNA and microRNA microarrays. Furthermore, bioinformatics analyses (including molecular function, biological processes, cellular components, KEGG pathway, and predicted target genes) were performed on mRNA or miRNA that displayed altered expression following RILI. The findings of the current study provide novel insight into the molecular mechanisms mediating radiation-induced damage and repair in the rat lung.

## Materials and methods

### Animals

Adult male Wistar rats (Shanghai Laboratory Animal Center, China) aged 7-8 wks and weighing 180-200 g was used in all experiments. All animal experiments were carried out according to the guidelines of the Chinese Council on Animal Care and approved by Nanjing Medical University Committees on Animal Experimentation. The rats were divided into two experimental groups: irradiated group (n = 24), sham-treated control group (n = 24). Rats were randomly selected (8 for control or irradiated groups) at the start of the experiment and sacrificed at each time point (3, 12 and 26 wks PI). No animals had to be prematurely sacrificed due to sickness.

### Lung irradiation protocol and follow-up

The rats were anesthetized prior to irradiation by an intraperitoneal injection of Ketamine hydrochloride (1-2 mg/kg). Hemithoracic irradiation was performed to the right lung with a single dose of 20 Gy using 6 MV x-rays (Varian 23EX Linear Accelerator, USA). A dose rate of 5 Gy/min was applied, based on a pilot study and previous reports [11]. The left thorax as well as the rest of the body was shielded with 3 mm of lead. Correct positioning of the fields was controlled for each individual rat using a therapy simulator (Huestis∙Cascade Simulator, Bristol, USA). The control rats were handled in the same manner, but were not irradiated.

### Sampling

Rats were killed humanely at 3, 12 and 26 weeks after irradiation, along with controls. Following sacrifice, whole blood and sera were collected and stored at −80°C. Each lung (right and left) was divided into three pieces: one fixed in 10% formalin solution for histological analysis, one for microarray analysis, and one stored at −80°C.

### Histologic analysis

For histological examination, the lung tissue was fixed, embedded in paraffin wax, sectioned, and stained with haematoxylin and eosin (H&E) or Masson’s Trichrome. For H&E staining, we evaluated an edema of the interstitial and intra-alveolar, infiltration of inflammatory cells, interstitial mononuclear cells, and interstitial collagen. The amount of pulmonary interstitial collagen was determined by Masson’s Trichrome. Slides were scored blindly and randomized and damage was assessed using each scoring method as previously described [15].

### RNA isolation and quantification

Total RNA was isolated from the right lung using Trizol reagent and RNeasy Mini Kit (Qiagen, Germany). RNA quality was assessed and confirmed using the Agilent 2100 Bioanalyzer (Agilent, U.S.A). The RNA samples were used for miRNA microarray, mRNA microarray, and qRT-PCR experiments. To avoid differences between individuals, 5 mg of total RNA isolated from 8 rats euthanized after irradiation (3, 12, 26 wks at each time point) and 8 control rats were pooled into a single sample for microarray experiment. Each pool hybridized repeat two times.

### Whole genome microarray analyses

Gene expression profiling was performed using the Agilent Whole Rat Genome 4 × 44K oligo microarray, for analysis of over 41,000 transcripts, according to the manufacturer’s instruction (all probes being printed in triplicate). Agilent’s Feature Extraction Software was used for array image analysis and the calculation of spot intensity measurements, which are considered to be raw data for the purposes of this study. The raw data files which contain “Processed Signal” were processed using the limma package developed within the Bioconductor project in the R statistical programming environment [16-18]. After log transformation, the data normalized using “quantile” method. Completely expressional data results were filtered, and the processed data was used to statistically analyze the data with absolute log2 fold change value cut off at 1 Benjamini-Hochberg correction were applied to obtain the false discovery rate (FDR).All steps from RNA amplification to the final scanner output were conducted by a private contractor (Shanghai OEBiotech.Co., Ltd, Shang hai, China). All data are MIAME (Minimum Information about a Microarray Experiment) compliant [19] and have been deposited in the EMBL/EBI Array Express database, (http://www.ebi.ac.uk/arrayexpress/).

### miRNA microarray analysis

The miRNA microarray analysis was performed by Phalanx Biotech Group (Hsinchu, Taiwan). Mouse & Rat miRNA OneArray microarrays (Phalanx Biotech Group, Palo Alto, CA) were used, and all probes being printed in triplicate. The Cy5 fluorescent intensities of each spot were analyzed by GenePix4.1 software (Molecular Devices). The signal intensity of each spot was processed by R program (http://www.r-project.org/) using the following 2 packages: limma (http://www.bioconductor.org/packages/release/bioc/html/limma.html) and genefilter (http://www.bioconductor.org/packages/release/bioc/html/genefilter.html). The fine signals (flag = 0) were extracted and processed by log2 transformation, quartile normalization method and ANOVA test. Individual genes with 2-fold difference in normalized expression and statistical significance in Welch t-test (P < 0.05) between control and irradiated group were identified as differentially expressed genes. All data are MIAME (Minimum Information about a Microarray Experiment) compliant [19] and have been deposited in the EMBL/EBI Array Express database, (http://www.ebi.ac.uk/arrayexpress/).

### Integrated analysis of miRNA and mRNA expression profiles

Integrated analysis of the miRNA and mRNA expression profiles were carried out using the lists of differentially expressed miRNA and mRNA. The predicted mRNA targets of each of the miRNAs that were differentially regulated were obtained using Target Scan version 4.0. A two-tailed Fisher’s Exact Test was conducted for each differentially regulated miRNA to determine whether the number of predicted target mRNAs that were differentially regulated was higher than would be expected by chance (P < 0.05). The Fisher Exact test was conducted for each miRNA using both down-regulated and up-regulated mRNA gene lists. The Agilent identifiers of the FDR significant probes were uploaded and mapped to genes in the Database for Annotation, Visualization and Integrated Discovery (DAVIDv6.7, http://www.ncbi.nlm.nih.gov/pmc/articles/PMC1933169/) for functional annotation. All available functional categories were considered, including Gene Ontology and KEGG Pathways (http://www.genome.jp/kegg/pathway.html). Network analysis was performed using Cytoscape (http://www.ncbi.nlm.nih.gov/pubmed/14597658).

### Real-time quantitative RT-PCR of mRNA and miRNA

Validation of differential gene expression was performed for a number of genes that were differentially expressed between irradiation and control groups. For mRNA RT-qPCR, total RNA was extracted and reverse transcribed to cDNA using the QuantiTect Reverse Transcription Kit (Qiagen). PCR was performed using the SYBR Green PCR Kit (Qiagen). A panel of PCR primers specific to the 4 representative genes that were up and down regulated was synthesized by Invitrogen (Shanghai, China). Primers used for qRT-PCR were listed in S4 supporting information. For miRNA RT-qPCR (let-7i and mir-21), experiments were carried out with the miScript Reverse Transcription and SYBR Green PCR Kit (Qiagen) according to manufacturer’s instruction. The reactions for miRNA and mRNA were automated by a 7900HT Real-Time PCR. Each PCR reaction was performed in triplicate. All calculations and analyses were performed using SDS RQ Manager 1.1 software using the 2^−ΔΔ^Ct method [20].

### Cell culture and transfection

Rat lung type II pneumocyte cells obtained from PriCells (Wuhan, China) were routinely cultured in DMEM medium containing 10% fetal bovine serum, at 37˚C with 95% humidity and 5% CO_2_. Chemically synthesized RNAs, including scramble, let-7i/miR-21mimics and inhibitors, were obtained from GenePharma (Shanghai, China). For transfection, cells were grown in 6-well culture plates to 70-80% confluence. The cell lines transiently transfected with miRNA mimics or inhibitors (0.5 μg) using Entranster™ -R transfection reagent (Engreen Biosystem, Beijing, China) according to the manufacturer’s instructions. The control mimics (50 nM) or control inhibitor (100 nM) was used as a control. After transfection for 48 h, the cells were harvested for further experiments. A 5-Cy3 negative control miRNA (Ribobio) was used for the measurement of transfection efficiency.

### Statistical analysis

Data is expressed using the mean and the standard deviation. Independent sample t test was performed to identify significant differences between control and treatment groups using SPSS 17.0 software. A p value of less than 0.05 was considered statistically significant.

## Results

### Histological changes in RILI

**Figure 1:**
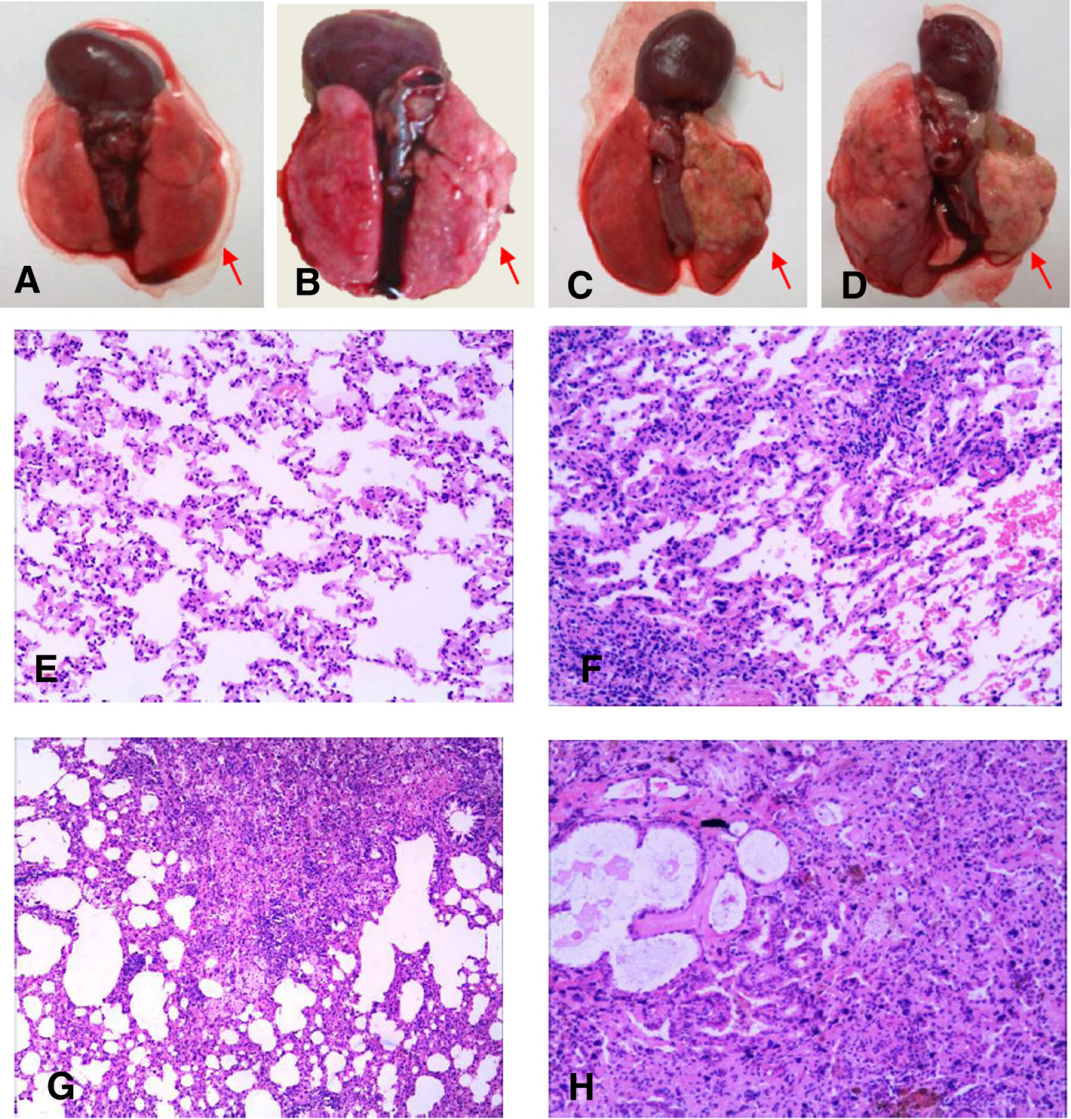
**Photomicrographs illustrating the histological response of lungs of rats irradiated with 20 Gy after a post-exposure period of approximately 26 weeks (H&E, 100X).** Gross examination of lungs at autopsy: **(A)** normal lung in non-irradiated rat; **(B)** 3 week; **(C)** 12 week; **(D)** 26 week; **(E)** Normal lung parenchyma and vessels in controls. **(F)** The third week after radiation shows the visibility of interstitial inflammatory edema and intra-alveolar hemorrhage; **(G)** The 12th week after radiation shows diffuse increase in alveolar septal thickening, vascular wall thickening and sclerosis, with focal necrosis and subsequent organization; **(H)** The 26th week after radiation shows fibroblast proliferation and increased collagen accumulation in the interstitial, as well as in the intra-alveolar space, and widespread loss of tissue architecture. (The right lung are indicated with arrowheads).

**Figure 2:**
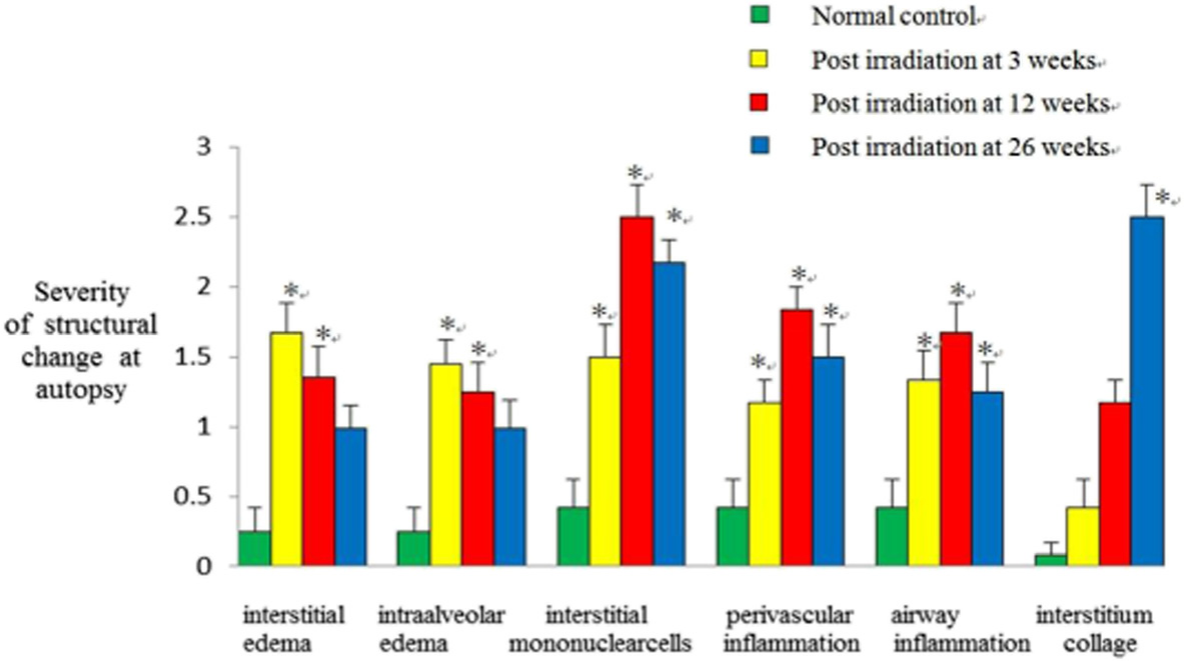
**Semi-quantitative assessment of morphologic changes following thoracic irradiation in autopsied specimens.** For H&E staining, we evaluated an edema of the interstitial and intra-alveolar, infiltration of inflammatory cells, interstitial mononuclear cells, and interstitial collagen at each time point. Data are expressed as means ± SE (n = 8). *p < 0.05 in 2-tailed student’s t test when compared to non-irradiated controls.

In the irradiated rats, unequivocal differences between the right (irradiated) and the left (non-irradiated) lung were observed (Figure 1A-D). Three weeks post irradiation, the biopsy tissue in all the irradiated animals showed multifocal minor alveolar reactive changes (minimal edema and minimal to mild increase of cellularity in alveolar walls, interstitial inflammation, enlargement and atypical of type II pneumocyte). These changes were more accentuated and frequent, with the visibility of the alveolar and vascular wall thickening, sclerosis, focal necrosis and subsequent organization by 12 weeks post irradiation. These alterations were increasingly visible until 26 wks when fibroblast proliferations, increased collagen accumulation in the interstitial as well as in the intra-alveolar space, widespread loss of tissue architecture were evident (Figure 1E-H). Significant differences between groups in scores were observed when these injuries were evaluated according to Downing *et al.*[15] by light microscopy at each time point (Figure 2). Additionally, in irradiated rats, the left lung showed minimal edema and minimal to mild interstitial inflammation, without fibrosis.

### mRNA expression profiling

**Figure 3:**
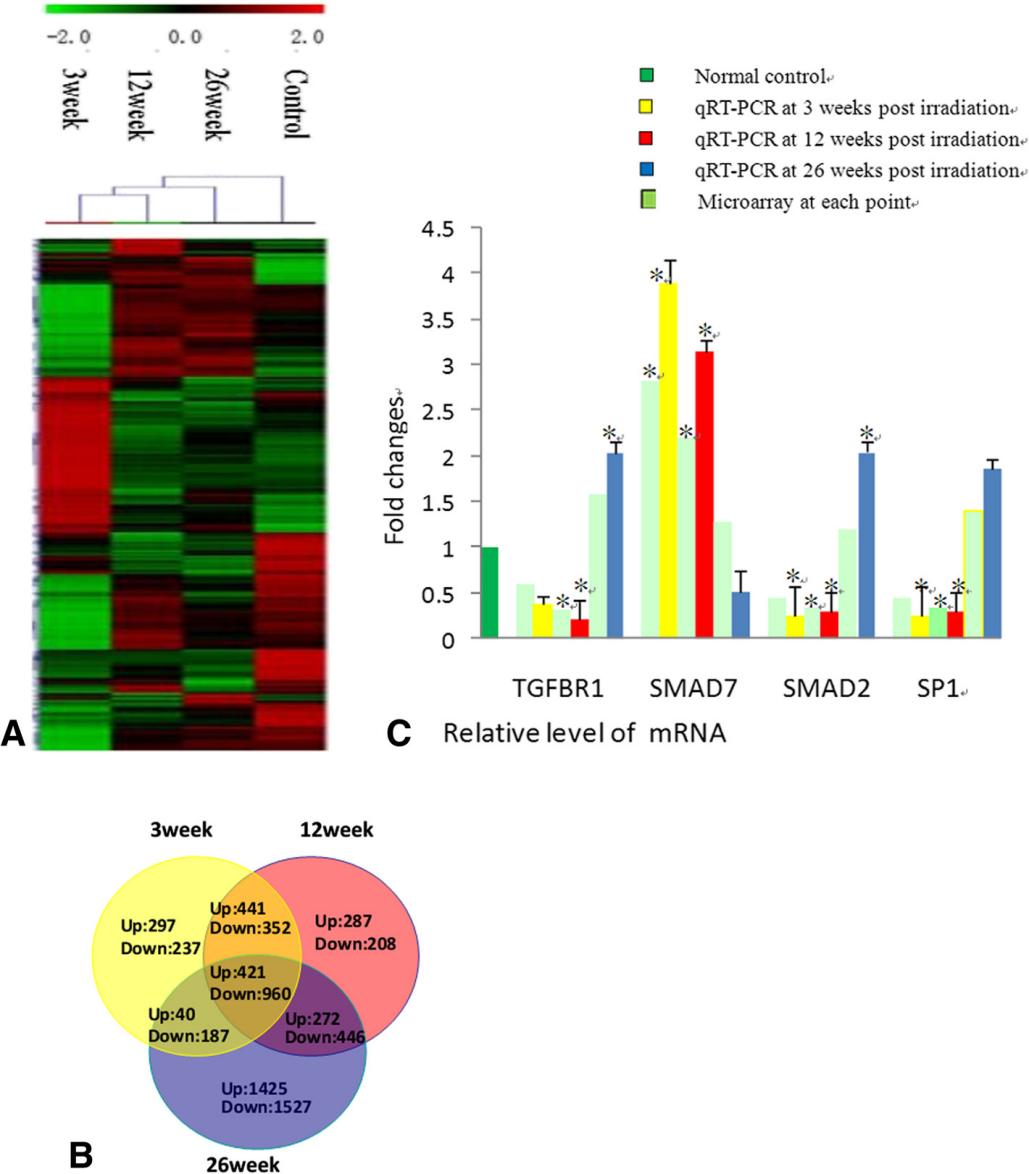
**Analysis of mRNA expression in the rat lung after irradiation. (A)** Unsupervised hierarchical clustering of differentially expressed mRNAs in early (3w), middle (12w) and late (26w) stages of RILI. Red indicates higher expression and green indicates lower expression in irradiated lung. Black means no expression difference. The inset above represents color scales used in the cluster map. **(B)** Overlaps of co-regulated mRNAs (greater than a 2-fold difference) across various post-irradiation time courses. The relative levels of individual mRNA transcripts with 2-fold difference and statistical significance by Welch t-test (P < 0.05), as compared with that of a non-irradiated control rat, were identified with differentially expressed mRNAs. Data shown are real numbers of up-regulated (Up) and down-regulated (Down) mRNAs. The numbers in the overlapped areas represent the number of differentially transcribed mRNAs shared by two or three groups. **(C)** qRT-PCR and microarray results of mRNA (TGFBR1,SMAD7,SMAD2,SP1) expression level at each post-irradiation time point. Each reaction was performed in triplicate and the average CT was used for RQ calculation. *p < 0.05, in 2-tailed student’s t test when compared to non-irradiated controls.

The significant different tissue ultra-structure of the irradiated rat lung compared to the control groups suggests that it harbors an important molecular driving force, which navigates its developmental process. In an effort to reveal the mechanisms essential for this process, greater than 20,000 rat genes and transcripts were investigated and the irradiated and control groups were compared. 2183 of the genes were found to be significantly up-regulated and 2917 to be significantly down-regu -lated (≥2.0 fold and P ≤ 0.05) in the irradiation group compared with control. Unsupervised clustering analysis (http://www.ncbi.nlm.nih.gov/pubmed/12613259) of the expression profiles revealed a distinct mRNA signature during RILI at each time point. Notably, analysis of mRNA expression revealed that 1199,1421,2158 up-regulated mRNAs, while1936,1966,3120 down-regulated mRNAs in the early (3w), middle (12w) and late (26w) stages of RILI (Figure 3A-B). The expression of selected differentially expressed genes was verified with quantitative RT-PCR (Figure 3C). It was obvious that many genes were differentially expressed in the early (3w), middle (12w) and late (26w) stages of RILI.

### Functional classification of differently expressed genes

In order to investigate the potential functional relevance of the differently expressed genes in RILI development process, we performed functional classification analysis of significantly regulated genes using the DAVID, and searched for significantly enriched biological processes and pathways. Gene Functional Classification assigns three major gene ontologies: molecular function, biological processes, and cellular components (Additional file 1: Figure S1A, B, C, D supporting information).

Molecular function analysis found that the 5100 differentially expressed genes were predominantly related to calcium ion binding, ion binding in RILI development process. However, cytokine and chemokine activity, etc. were ranked among the significantly over-represented pathways in the early (3w) stage of RILI-enriched genes; ion and substrate specific channel activity, protein dimerization activity, etc. were ranked in the middle (12w) stage; nucleoside binding, etc. were ranked in the late (26w) stage.

Analysis of the biological processes revealed the 5100 differentially expressed genes to be predominantly associated with inflammatory response, biological adhesion in RILI development process. However, immune and defense response were ranked as the significantly over-represented pathways in the early stage; wound healing, blood vessel development, etc. were ranked in the middle stage; cell migration, positive regulation of cell communication, etc. were ranked in the late stage.

Cellular component analysis identified the 5100 differentially expressed genes to be primarily related to plasma membrane part, cell projection in RILI. However, cytoskeletal part and vesicle were ranked as the significantly over-represented pathways in the early stage; membrane raft, apical plasma membrane, etc. were ranked in the middle stages; endoplasmic reticulum, endomembrane system, etc. were ranked in the late stages.

To define the biological pathways potentially associated with irradiation in the rat lung, microarray data were analyzed using DAVID, KEGG pathway analysis. It revealed that the genes whose expression was significantly altered in RILI development process are associated with cytokine-cytokine receptor interaction, Chemokine signaling, etc. However, p53 signaling, cell adhesion molecules etc. were ranked as the significantly over-represented pathway in the early stage; B cell receptor signaling, VEGF signaling, etc. were ranked in the middle stage; Toll-like receptor signaling, TGF-β signaling, etc. were ranked in the late stage.

### miRNA expression profiling

**Figure 4:**
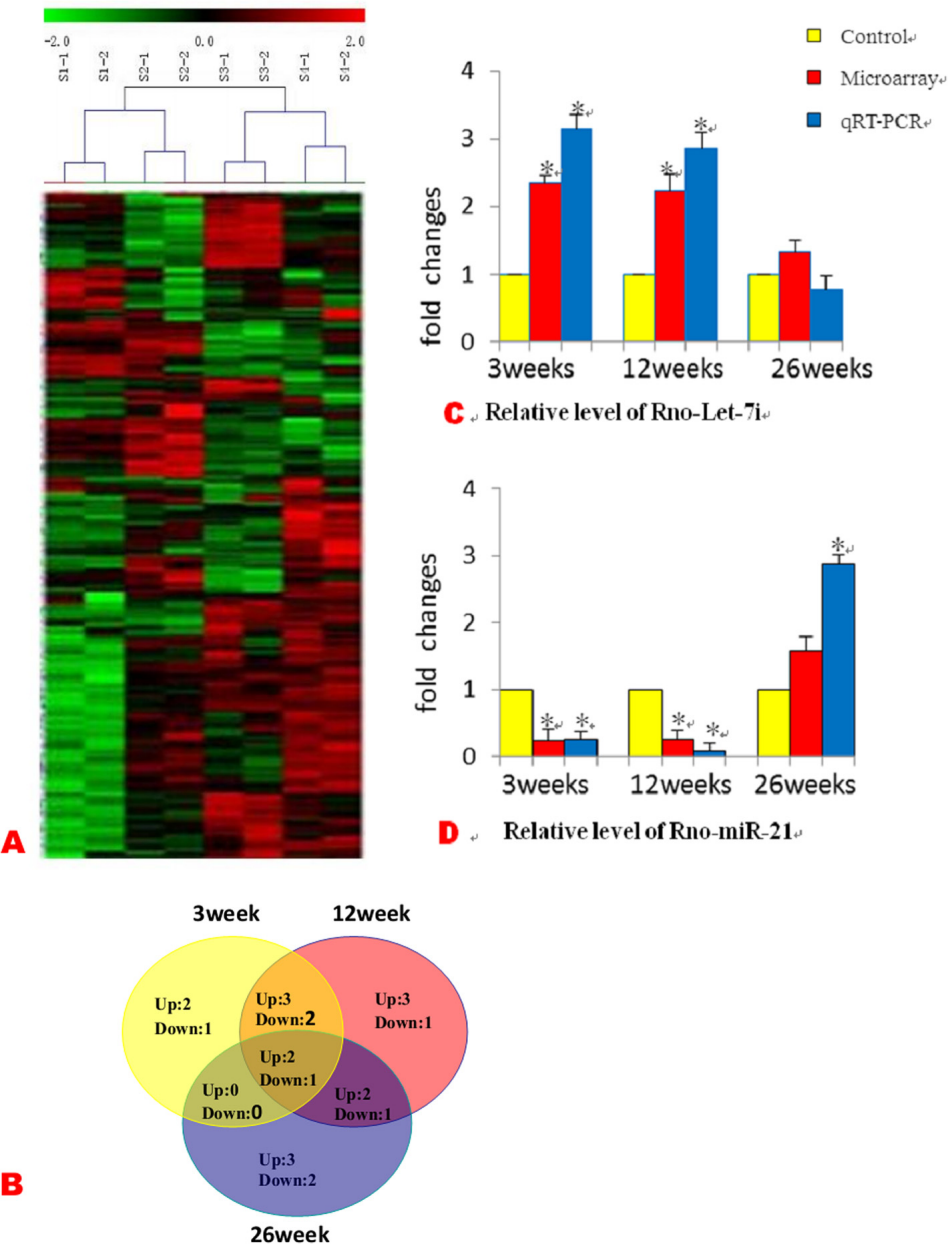
**miRNA expression in the rat lung after irradiation. (A)** Unsupervised hierarchical clustering of differentially expressed miRNAs in early (3w), middle (12w) and late (26w) stages of RILI. Red indicates higher expression and green indicates lower expression in irradiated lungs. Black means no expression difference. **(B)** Overlaps of co-regulated miRNAs (>2.0 fold) across various post-irradiation time courses. Data shown are real numbers of up-regulated (Up) and down-regulated (Down) miRNAs. The numbers in the overlapped areas represent the number of differentially transcribed miRNAs shared by two or three groups. **(C, D)** qRT-PCR and microarray results of miRNA (Rno-miR-21and Rno-Let-7i) expression level at each post-irradiation time point. Results are expressed as relative quantifications (RQ) of the miRNA expression level in each sample relative to non-irradiated controls, and normalized to U6 snRNA. Each reaction was performed in triplicate and the average CT was used for RQ calculation. *p < 0.05, in 2-tailed student’s t test when compared to non-irradiated controls.

A microarray platform optimized for the analysis of 387 rat miRNAs was used to analyze and compare the pattern of miRNA expression between irradiated and control rat. Expression of 23 miRNAs were found to be significantly different between the four groups (P ≤ 0.05) comprising of 15 up-regulated and 8 down-regulated (irradiated group at each time point vs. sham-treated control group). To confirm the microarray results, qRT-PCR was performed on two randomly selected differentially expressed miRNAs (let-7i and miR-21). The selected miRNAs exhibited expression levels that are consistent with the microarray results, indicating the reliability of the microarray data (Figure 4C/D). Unsupervised clustering analysis of the expression profiles revealed a distinct miRNAs signature during RILI and demonstrated that the majority of the differentially expressed miRNAs were up-regulated in response to irradiation (Figure 4A/B).

### Integrated analysis of miRNA and mRNA expression profiles

Because most mammalian miRNAs are intragenic and transcribed as part of their hosting transcription units, we hypothesized that the expression profiles of mature miRNAs and their host genes are directly correlated. miRNA expression was compared with their host mRNA expression to see whether they were co-expressed. The list of rat intragenic miRNAs and corresponding host genes was retrieved from miRBase (Release16.0, 2009). Analysis of the identified miRNAs revealed that 7 up-regulated miRNAs had 90 down-regulated targets, while 4 down-regulated miRNAs had 50 up-regulated target mRNAs in the early (3w) stages of RILI;10 up-regulated miRNAs had 180 down-regulated targets, while 4 down-regulated miRNAs had 65 up regulated target mRNAs in the middle (12w) stages; 7 up-regulated miRNAs had 95 down-regulated targets, while 4 down-regulated miRNAs had 40 up-regulated target mRNAs in the late (26w) stages.

To determine whether different miRNAs within a signature interact with the same target genes, we performed network analysis using Cytoscape, a software platform for the visualization of complex interaction networks. We performed network analyses using top 10 identified miRNAs (up-regulated: let-7i, let-7c, let-7a, miR-124, miR -145, miR-143, miR-34a, miR-466; down-regulated: miR-21, miR-146b) to predict their potential target transcripts. We found that these mRNA targets are involved in cytokine-cytokine receptor interaction, chemokine, p53, TGF, and MAPK signaling pathway, etc. in the early, middle and late stages of RILI development process. Taken together, our results suggest that a small group of miRNAs maybe coordinately regulates a pathway transcriptome in response to radiation. As shown in Additional file 1: Figure S2 (supporting information), most of the transcripts identified by network analysis are associated with more than one miRNA, suggesting multiple miRNA-mRNA interactions combinatorial effect in gene regulation by coexpressed endogenous miRNAs in RILI.

### Validation of the functional pathways analysis

To further test whether the functional pathways associated to the identified miRNAs could be reproduced experimentally *in vitro*, we performed validation experiments testing miRNAs involved in the TGF-β signaling pathway (miR-21/SMAD7, let-7i/TGFBR1). No functional data of these miRNAs have been reported in RILI. To determine the cell type-specific localization of miR-21 and let-7i, fluorescence-based in situ hybridization (ISH) was conducted on formalin-fixed, paraffin-embedded (FFPE) tissues sections of rat lungs using LNA (Locked Nucleic Acid) anti-miR-21, anti-let-7i and scrambled control probes. Let-7i was primarily detected in the cytoplasm of alveolar cells in irradiated lungs (Additional file 1: Figure S3A supporting information). MiR-21 was expressed in the cytoplasm of most alveolar cells and to a lesser extent expressed in the cytoplasm of fibroblast/myofibroblast cells in lung tissue (Additional file 1: Figure S3B supporting information). To determine if miR-21 and let-7i were indeed expressed in pulmonary alveolar cells in irradiated lungs, immunohistochemistry was performed along with ISH and demonstrated that miR-21 and let-7i expression were primarily colocalized with that for TTF1 (Thyroid transcription factor1), suggesting that alveolar cells were the main source of the increased miR-21 and let-7i levels present in irradiated lungs. (Additional file 1: Figure S3C/D supporting information).

Based on the miRNA-mRNA profiling integration analysis, miR-21, let-7i expression were negatively associated with TGF-β signaling genes such as SMAD7, TGFBR1, suggesting that these miRNAs could regulate the TGF-β signaling in RILI. TGF-β signaling plays important roles in fibrotic disorders such as diabetic nephron -pathy, Crohn’s disease, rheumatoid arthritis, radiation-induced fibrosis, and myocarditis [21,22]_._ The TGFBR1 gene, which mediates the action of TGF-β, is a predicted target gene of the let-7/miR-98 family according to TargetScan 4.2 (containing conserved sites for let-7a-g and i, and for miR-98). SMAD7, which is an inhibitory Smad that negatively regulates Smad2 and Smad3 activation, is a negative regulator of TGF-β signaling, and is a predicted target gene of miR-21. Our results showed a negative correlation between the expression of let-7i, miR-21 and TGFBR1, SMAD7, respectively during RILI, which was evident let-7i expressed higher in the early and middle stages over the control, and then expressed lower in late stage. However, miR-21 expressed higher at each time point (Figure 4C/D). In order to examine whether let-7i, miR-21 may directly regulate TGFBR1 and SMAD7 expression, we overexpressed or inhibited miR-21and let-7i miRNA in the rat lung type II pneumocyte cells, and measured the expression of TGFBR1, SMAD7, SMAD2, SP1 (Specificity Protein 1) mRNA and protein.

**Figure 5:**
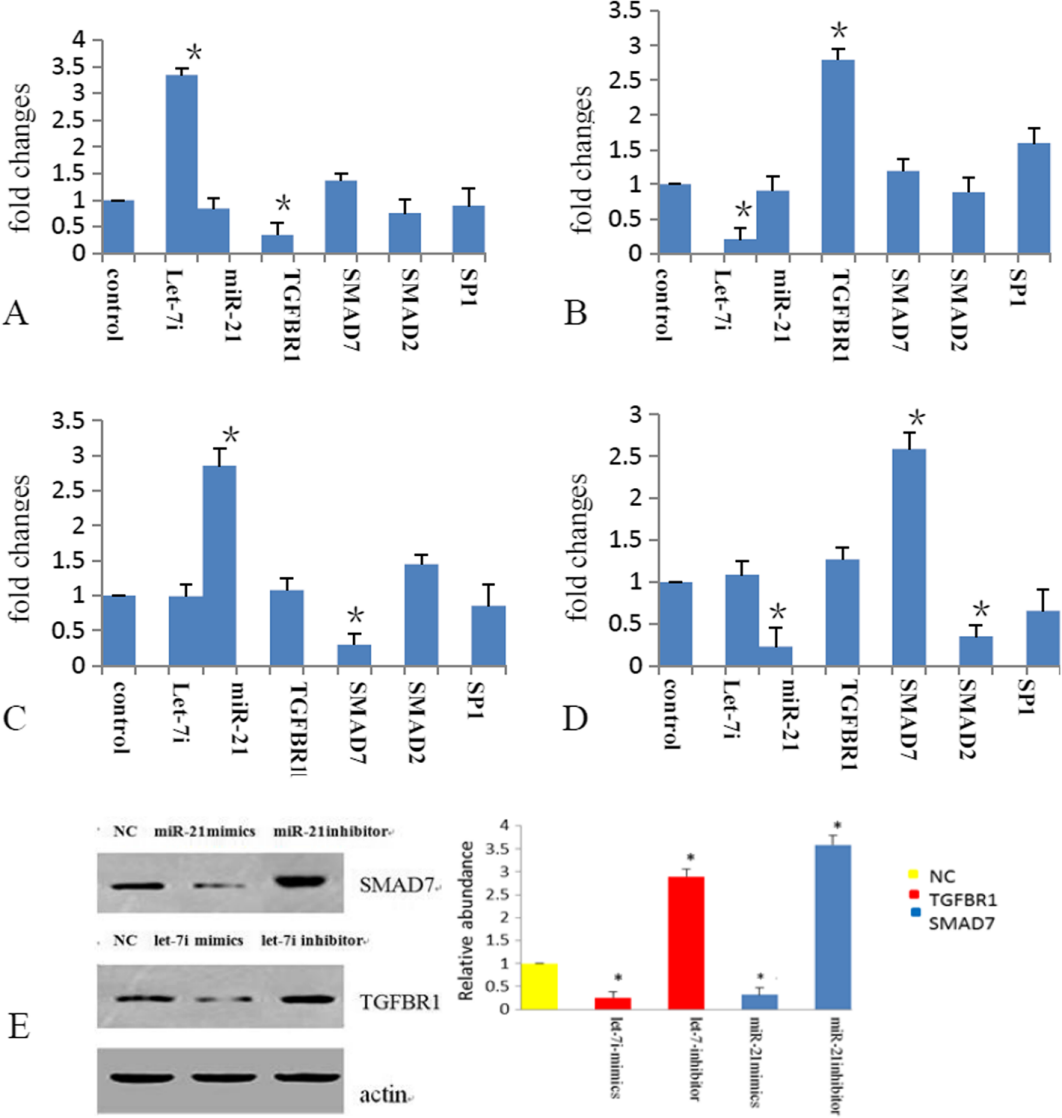
**Prediction and confirmation of the target genes predicted for both let-7i and miR-21in rat lung type II pneumocyte cells.** The cells were co-transfected with a negative control, let-7i and miR-21 mimics or inhibitors, respectively; **(A/B)** The relative mRNA expression level of the target genes (TGFBR1/TGF-β pathway) of let-7i mimics and inhibitors in cultured rat lung type II pneumocyte cells 48 h after transfection. **(C/D)** The relative mRNA expression level of the target genes (SMAD7/ TGF-β pathway) of miR-21 mimics and inhibitors in cultured rat lung type II pneumocyte cells 48 h after transfection. **(E)** Western blot analysis of TGFBR1/SMAD7 protein level in rat lung type II pneumocyte cells transfected as described in A for 72 hrs. Quantification of the bands’ intensity was shown on the right. All experiments were repeated at least three times independently. The results are presented as means ± S.E.M. *P < 0.05.

Using transient transfection, we up-regulated let-7i and miR-21 by miRNA mimic, or down-regulated let-7i and miR-21 by miRNA inhibitor in the rat lung type II pneumocyte cells. Based on fluorescence microscopy we found that miRNA fragments were successfully transfected into cells with the transfection efficiency >80% (data not shown). Furthermore, miRNA expression of up and down-regulation after transfection was confirmed by qRT-PCR. Overexpression of let-7i or miR-21was capable of inhibiting the expression of TGFBR1 or SMAD7 mRNAs and protein, and inhibition of let-7i or miR-21 promoted the expression of TGFBR1 or SMAD7 mRNA and protein, and inhibited SMAD2 mRNA (p < 0.05) (Figure 5 and Additional file 1: Figure S4 supporting information).

## Discussion

### Histological changes in RILI

In the present study, we have shown a sequence of morphological changes in the lungs of rat from the early to late stage following a single dose irradiation with 20 Gy to the right lung, a dose known to be high enough to cause radiation pneumonitis relating to fibrogenesis. Overall, the histopathological changes observed in the irradiated rats were not different with previously published results using other lung irradiation models [9,10]. The development of lung damage after radiotherapy is a continuous process that can be attributed to radiation damage in parenchymal cells. In general, it is the previously mentioned cell types, specifically the inflammatory, fibroblastic, and epithelial cells, that appear to play the most critical roles in radiation-induced pulmonary pathogenesis, and therefore it is likely that these cells and their response to injury, as well as the participating signaling molecules will provide us with the most promising information for drug intervention [23]. In contrast, the endpoints examined did not appear to demonstrate that putative inflammatory mediators released in the irradiated right lung evoked any significant change in the non-irradiated contralateral left lung. This is in line with a study by Baumann *et al.*[24] in minipigs indicating that irradiation of a small lung volume with high fibrogenic doses did not affect the radiation dose-response relationship for development of fibrosis in distant parts of the ipsilateral lung.

### mRNA expression profiles in the RILI

In our study, thousands of genes been significantly up regulated or down -regulated during RILI development process. Based on gene ontology analysis of biological processes and pathways reveal the 5100 differentially expressed genes to be predominantly associated with response to wounding, inflammatory stimuli, and biological adhesion. These cellular events are associated with cytokine-cytokine receptor interaction, chemokine signaling, and focal adhesion in RILI development process. However, there exist significantly different biological processes and pathways between the irradiation and control groups at various post irradiation time points. Similarly, Alexandra *et al.*[25] used microarrays to define the radiation responses of alveolitis and fibrosis at the gene expression level. Pathway analysis revealed the expression of complement and of B-cell proliferation and activation genes to distinguish fibrosis from the alveolitis response and cytokine interactions and intracellular signaling differed between A/J and C3H mice. A genomic approach was used to identify specific pathways that likely contribute to the lung response to radiation as fibrosis or alveolitis in mice. Classically, RILI were deemed to be the consequence of cell loss in either the parenchymal or vascular compartments; however, they are now seen as the result of a complex, orchestrated interaction between multiple cell types, initiated and perpetuated through inter- and intra-cell signaling [26]. Further study on these differentially expressed genes in rat lung should be beneficial to better understand the cellular mechanism of RILI.

### Integrating miRNA and mRNA expression profiles in the RILI

Comparison of miRNA expression profiling between the irradiated and control group at various post irradiation time points revealed distinct expression patterns, which suggest that microRNAs may play an important role in the pathogenesis of RILI. To further investigated the role of miRNAs in RILI, we integrated miRNA with mRNA expression profiles and identified positively and negatively correlated miRNA/mRNA pairs. A total of 23 miRNAs and 5100 mRNAs were found to be differentially expressed in response to radiation, which the altered miRNAs and their 520 predicted mRNA targets displaying reciprocal levels of expression. These interactions appear to take place within cytokine-cytokine receptor interaction, chemokine signaling, focal adhesion, TGF-MAPK signaling, etc. Many of these interactions have been experimentally validated in other disease models. The negative correlation between miRNAs and predicted target mRNA expression identified in this study supports the hypothesis that miRNAs significantly modulate gene expression in response to radiation. Additionally, the regulatory effect of miRNAs that do not show negative correlation with their target genes may be masked by additional regulatory machineries, which remain uncharacterized.

We focused only on the miRNAs whose predicted target genes showed significant correlation with their cognate miRNA expression by our algorithm. In this study, the expressions of let-7i and miR-21 were altered in RILI lungs. They both participate in feed-forward loops that inhibit or amplify the TGF-β signaling in RILI. Thus, TGF-β confers opposite effects depending on the stages of RILI development, perhaps by the combined effect of different factors in each stage. Regulation of TGFBR1 by let-7 and SMAD7 by miR-21, which was suggested by our results, may further fine-tune the TGF-β signaling activity to the necessary level at each RILI developmental stage. A likely scenario, in the early stage, is that let-7 expressed higher and miR-21 level is lower, which inhibits TGF-β signaling that is necessary for lung regeneration. Whereas in the late stage, let-7 expressed at a relatively low level and miR-21 level is higher, which may allow an enhanced TGF-β signaling activity that is necessary for lung fibrosis. Taken together, the changes in let-7 and miR-21 expression level create an unopposed profibrotic balance in RILI development. Thus, the design of therapeutic interventions will require consideration of these complex interactions and application of system biology approaches may aid in the identification of the key points of intervention.

In summary, RILI does not develop gradually in a linear process. In fact, different cell types interact via cytokines in a very complex network. The complexity of radiation lung pneumopathy at the molecular and cellular level, however, indicates that switching off the whole procedure would require targeting of more than one molecular pathway. Although relatively little information is available concerning the biological function of miRNAs identified to date, we strongly suggest that miRNAs play an important role in modulating gene expression in the pathophysiological response to radiation in the rat lung. We are interested in further investigating how radiation modulates the transcription of those miRNAs and their predicted target genes in RILI.

## Competing interests

The authors declare that they have no competing interests.

## Authors' contribution

LX carried out most of the practical work and drafted the manuscript. JZ and SY contributed to analysis and interpretation of the data. QC, CS performed the animal experiment. JM performed part of the molecular biology and cell culture. JW, RS and QD participated in study design and modified the manuscript. All authors read and approved the final manuscript.

## Supplementary Material

Additional file 1: Figure S1 A. Lists of the top 20 molecular functions which were significantly enriched in differentially expressed genes between the irradiation and control groups at various post-irradiation time points. B. Lists of the top 20 biological processes which were significantly enriched in differentially expressed genes between the irradiation and control groups at various post-irradiation time points. C. Lists of the top 20 cellular components which were significantly enriched in differentially expressed genes between the irradiation and control groups at various post-irradiation time points. D. Lists of the top 20 KEGG pathways which were significantly enriched in differentially expressed genes between the irradiation and control groups at various post-irradiation time points. **FigureS2.** Functional analyses of differentially expressed predicted targets of significantly altered microRNAs. A: microRNA let-7i was up regulated; miR-146b and miR-21 were down regulated 3 weeks post-irradiation vs. the non-irradiated group. B: microRNAs let-7i, let-7a, let-7c, miR-34a, miR-124, miR-145, and miR-143 were up regulated; miR-21 was down regulated 12 weeks post-irradiation vs. the non -irradiated group. C: microRNAs miR-466b and miR-21 were up regulated; miR-146b was down regulated 26 weeks post-irradiation vs. the non-irradiated group. **Figure S3.** Reciprocal expression of TTF1 and let-7i, miR-21. **Figure S4.** Primers used for qRT-PCR. **Figure S5.** Western blot. Click here for file
