# Supplementary material for: Integrating microRNA and mRNA expression profiles in response to radiation-induced injury in rat lung
Source: Radiat Oncol. 2014 May 9;9:111. doi: 10.1186/1748-717X-9-111 (PMC4044330; doi:10.1186/1748-717X-9-111)
Supplement: Additional file 1: Figure S1 — A. Lists of the top 20 molecular functions which were significantly enriched in differentially expressed genes between the irradiation and control groups at various post-irradiation time points. B. Lists of the top 20 biological processes which were significantly enriched in differentially expressed genes between the irradiation and control groups at various post-irradiation time points. C. Lists of the top 20 cellular components which were significantly enriched in differentially expressed genes between the irradiation and control groups at various post-irradiation time points. D. Lists of the top 20 KEGG pathways which were significantly enriched in differentially expressed genes between the irradiation and control groups at various post-irradiation time points. FigureS2. Functional analyses of differentially expressed predicted targets of significantly altered microRNAs. A: microRNA let-7i was up regulated; miR-146b and miR-21 were down regulated 3 weeks post-irradiation vs. the non-irradiated group. B: microRNAs let-7i, let-7a, let-7c, miR-34a, miR-124, miR-145, and miR-143 were up regulated; miR-21 was down regulated 12 weeks post-irradiation vs. the non -irradiated group. C: microRNAs miR-466b and miR-21 were up regulated; miR-146b was down regulated 26 weeks post-irradiation vs. the non-irradiated group. Figure S3. Reciprocal expression of TTF1 and let-7i, miR-21. Figure S4. Primers used for qRT-PCR. Figure S5. Western blot. [file 1748-717X-9-111-S1.doc]

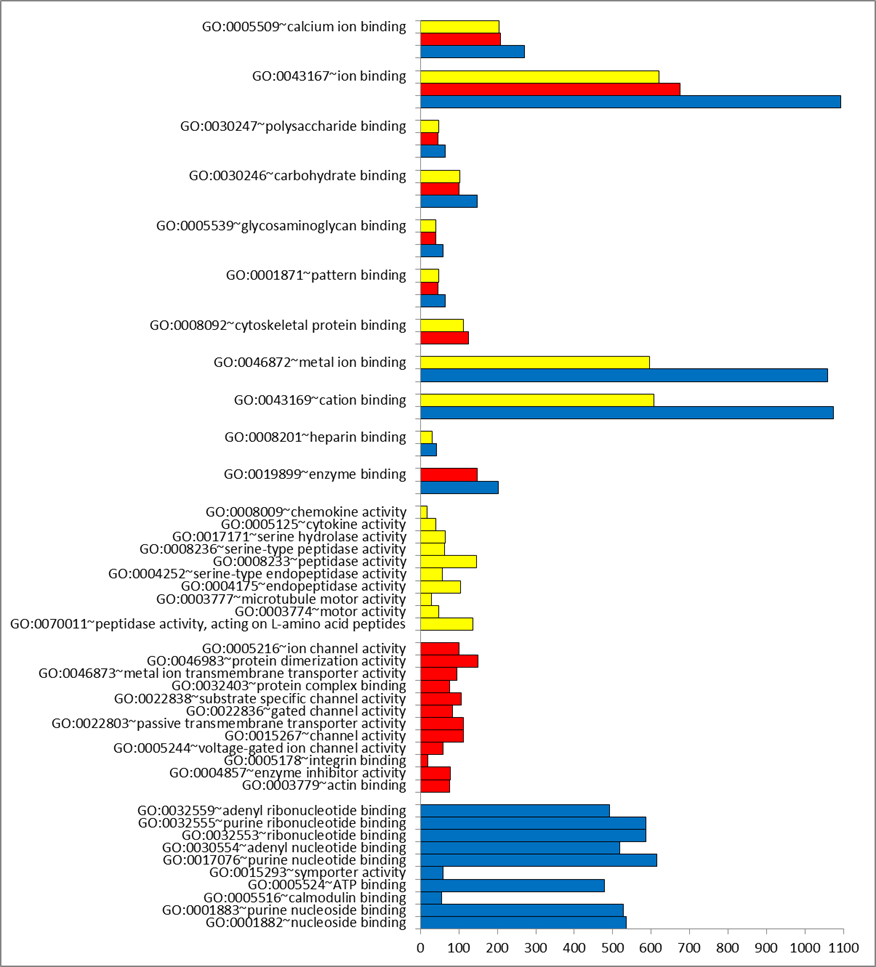


Fig.S1A. Lists of the top 20 molecular functions which were significantly enriched in differentially expressed genes between the irradiation and control groups at various post-irradiation time points. Yellow, red, and blue colors indicate early stages (3wk), middle stages (12wk), and late (26wk) stages of RILI development, respectively. Only categories with a p-value greater than 0.05 are included.


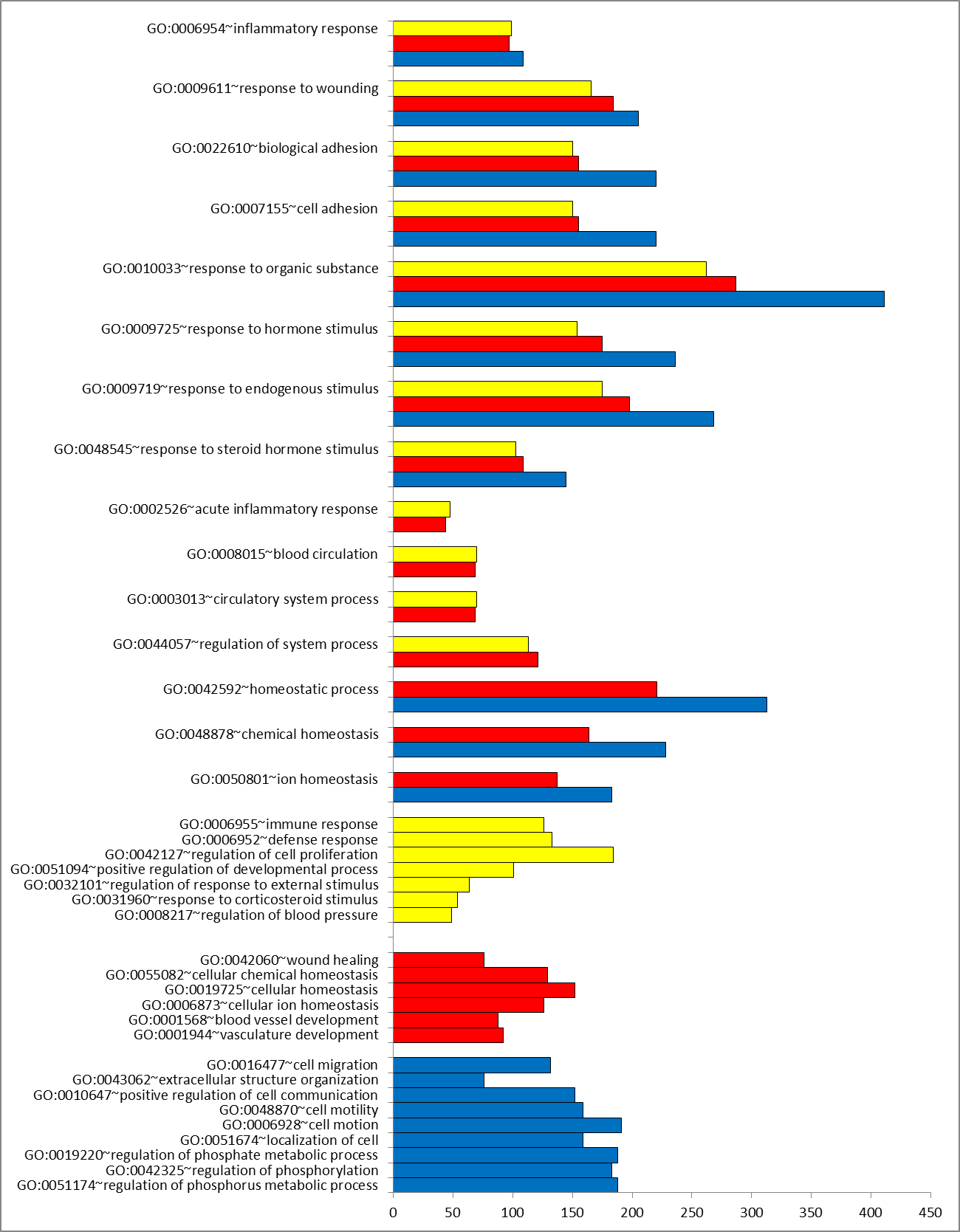


Fig.S1B. Lists of the top 20 biological processes which were significantly enriched in differentially expressed genes between the irradiation and control groups at various post-irradiation time points. Yellow, red, and blue colors indicate early stages (3wk), middle stages (12wk), and late (26wk) stages of RILI development, respectively. Only categories with a p-value greater than 0.05 are included.


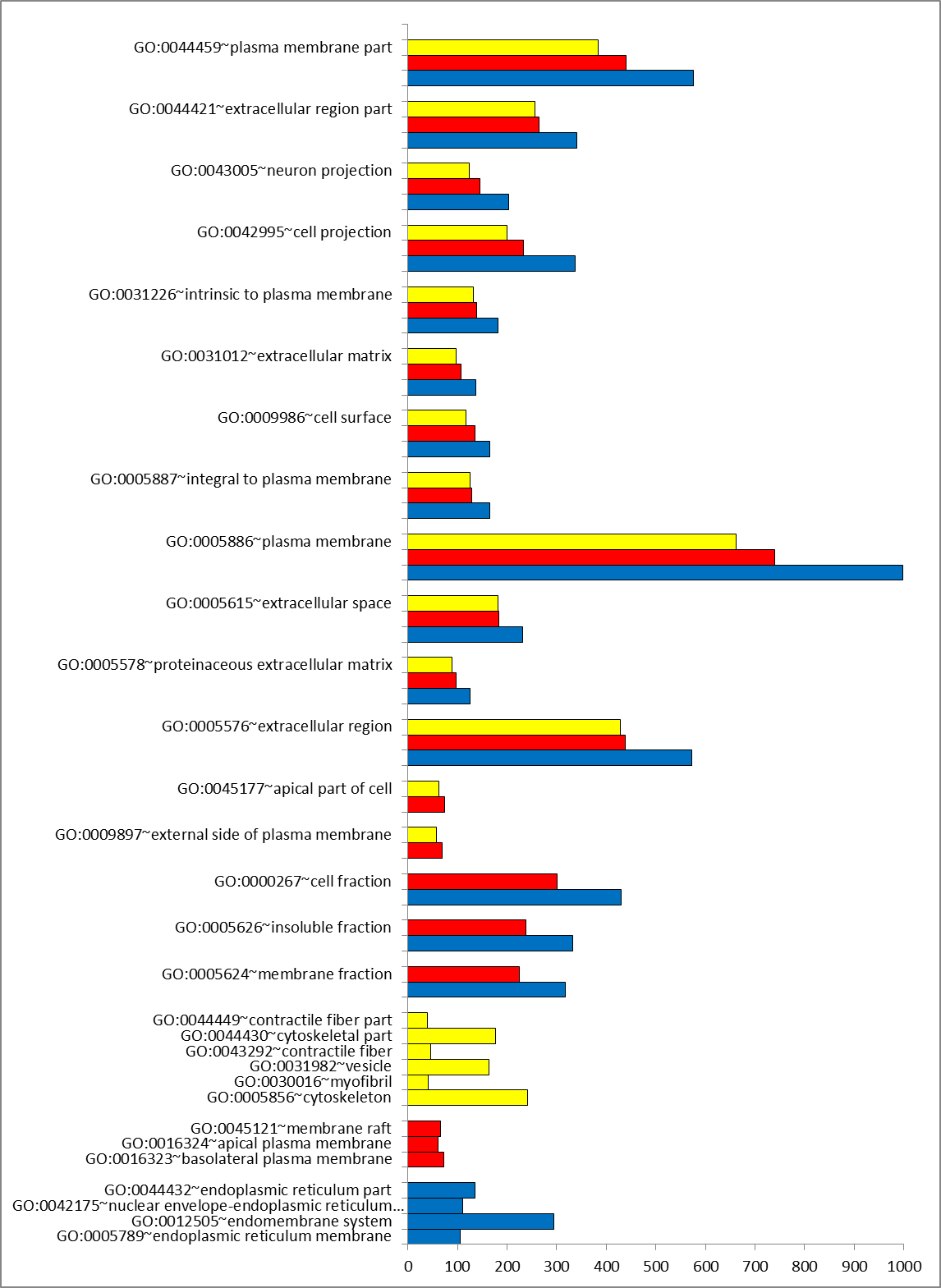


Fig.S1C. Lists of the top 20 cellular components which were significantly enriched in differentially expressed genes between the irradiation and control groups at various post-irradiation time points. Yellow, red, and blue colors indicate early stages (3wk), middle stages (12wk), and late (26wk) stages of RILI development, respectively. Only categories with a p-value greater than 0.05 are included.


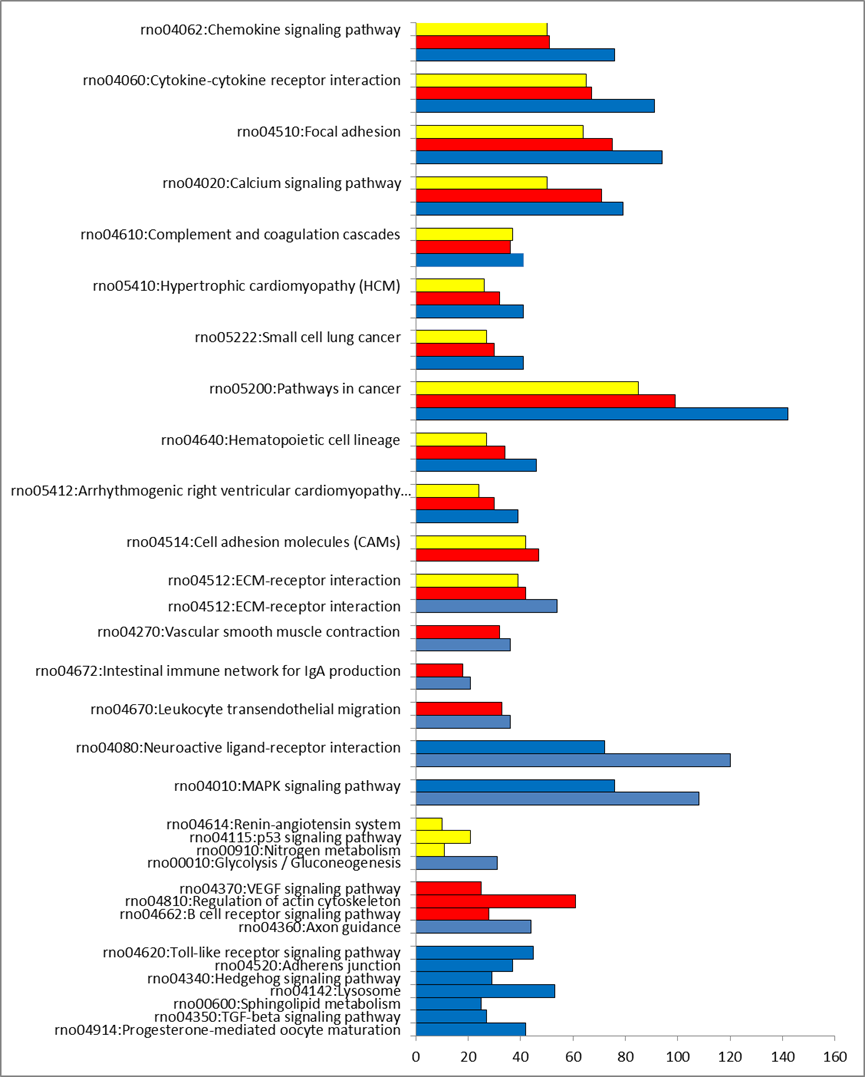


Fig. S1D. Lists of the top 20 KEGG pathways which were significantly enriched in differentially expressed genes between the irradiation and control groups at various post-irradiation time points. Yellow, red, and blue colors indicate early stages (3wk), middle stages (12wk), and late (26wk) stages of RILI development, respectively. Only categories with a p-value greater than 0.05 are included.


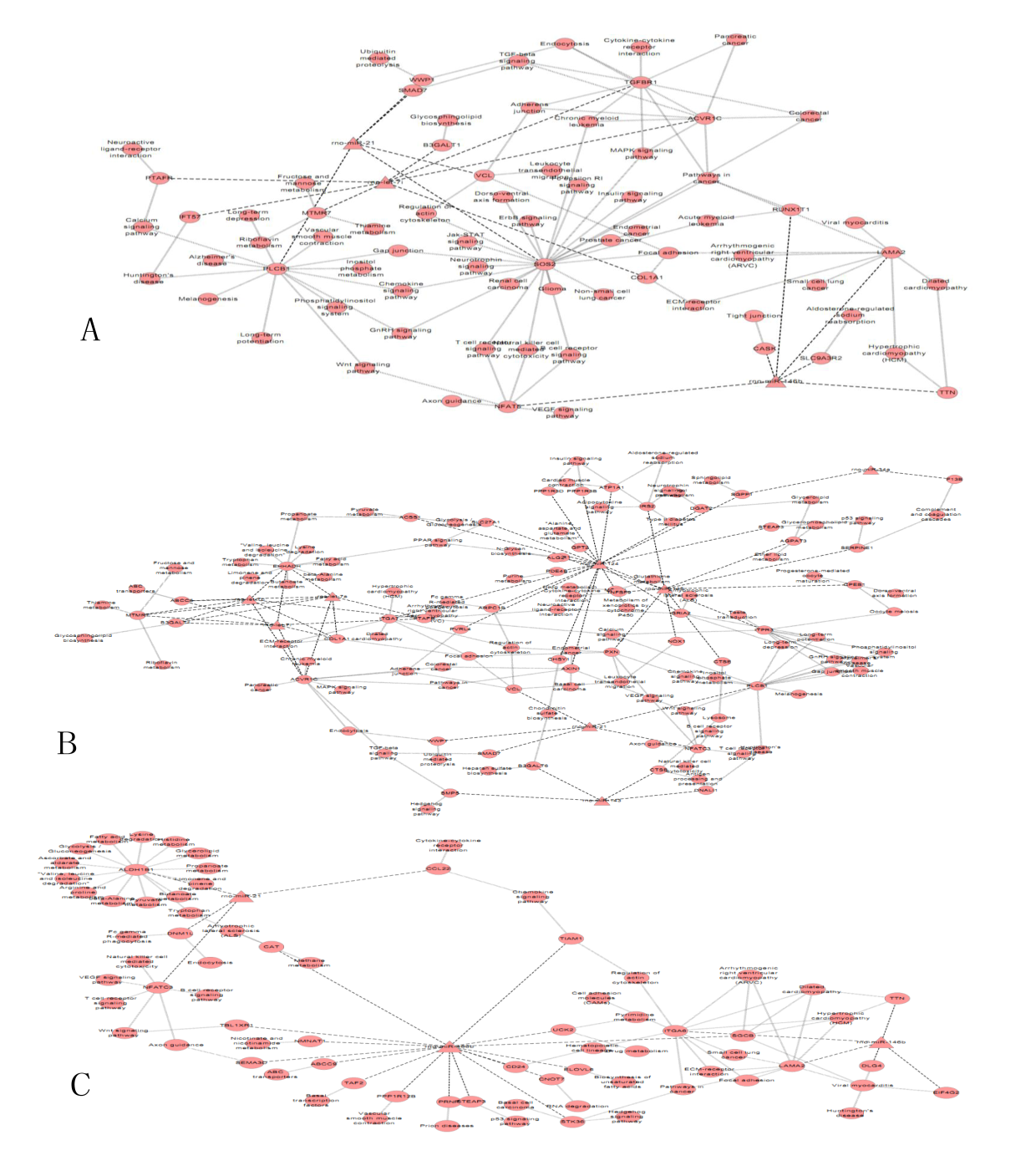


Fig.S2. **Functional analyses of differentially expressed predicted targets of signiﬁcantly altered microRNAs**. A: microRNA let-7i was up regulated; miR-146b and miR-21 were down regulated 3 weeks post-irradiation vs. the non-irradiated group. B: microRNAs let-7i, let-7a, let-7c, miR-34a, miR-124, miR-145, and miR-143 were up regulated; miR-21 was down regulated 12 weeks post-irradiation vs. the non -irradiated group. C: microRNAs miR-466b and miR-21 were up regulated; miR-146b was down regulated 26 weeks post-irradiation vs. the non-irradiated group. Their differentially expressed predicted targets are also shown. Signaling networks identiﬁed by Cytoscape software are based on signiﬁcant fold changes of the differentially expressed microRNAs. Solid lines between genes/microRNAs represent a direct interaction based on experimentally proven associations. Dashed lines represent predicted interactions (indirect interactions) based on experimental evidence.


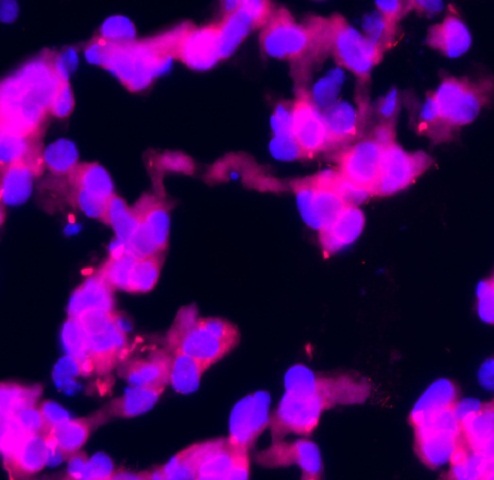

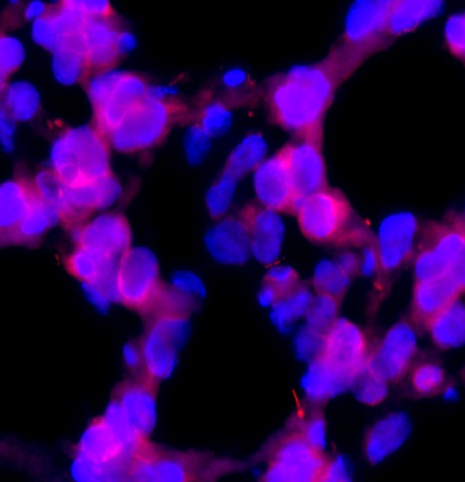


A:let-7i

B:miR-21

R


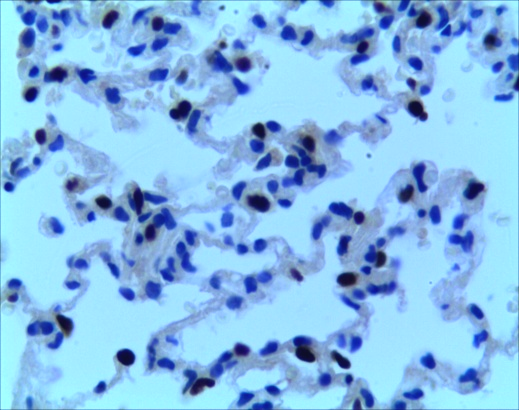

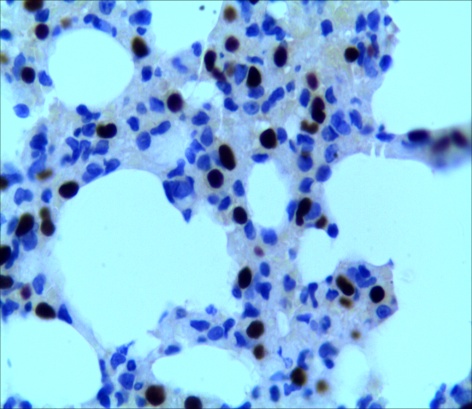


C

D

Fig.S3. Reciprocal expression of TTF1 and let-7i, miR-21. (A/B)The distribution of let-7i and miR-21 in rat lung tissue were assessed by in-situ hybridization. Paraffin-embedded sections of lung tissue were hybridized with a 5’ fluorescein -conjugated, LNA-modified DNA probes complementary to let-7i, miR-21. The combined images of cytoplasmic miR-21/let-7i highlighted with TRITC signal (red) and nuclear DNA counterstained with 4′, 6-diamidino-2-phenylindole (DAPI; blue), Magnification, 1000x. (C/D) The same paraffin-embedded lung tissue (A-C/B-D) was immunostained with TTF1 antibodies. TTF-1 is a tissue-specific transcription factor expressed in the type II pneumocytes of lung. Antibody binding was visualized with the substrate-chromogen DAB. TTF1 positive cells were stained brown in nucleus, 400x.

S4: Primers used for qRT-PCR:

| RAT-GAPDH –U | AGATGGTGAAGGTCGGTGTG |
| --- | --- |
| RAT-GAPDH –L | TCGTTGATGGCAACAATGTC |
| RAT-TGFBR1-U | 5' ATA TTC CCA ACA GAT GGC AGA G 3' |
| RAT-TGFBR1-L | 5' CTC CAT TGG CAT ACC AAC ATT C 3' |
| RAT-SP1-U | 5' CTC GTT CGG ATG AGC TTC AG 3' |
| RAT-SP1-L | 5' ATG AAA CGC TTA GGG CAC TC 3' |
| RAT-SMAD2-U | 5' AGT GTG TCA CCA TAC CAA GCA 3' |
| RAT-SMAD2-L | 5' CCT GTT GTG TCC CAC TGA TCT 3' |
| RAT-SMAD7-U | 5' ACT CCA GAT ACC CGA TGG AT 3' |
| RAT-SMAD7-L | 5' TTC ATC GGA GGA AGG TAC AG 3' |
| rno-let-7i-U | GTGAGGTAGTAGTTTGTGCTGTT |
| rno--21-U | GGCTAGCTTATCAGACTGATGTTGA |
| rno- U6-U | 5' GCT TCG GCA GCA CAT ATA CT 3' |

S5: Western blot

Treated cells in a six-well culture cluster were washed twice with ice-old phosphate-

buffered saline and then directly lysed in 200ul of 2× sodium dodecyl sulfate cell lysis buffer in each well. The lysates were boiled, centrifuged at 10 000 r.p.m. and then loaded onto a 12% sodium dodecyl sulfate–polyacrylamide gel electrophoresis gel. The samples were electrophoresed for 4 h and then transferred to a Millipore Immobilon transfer membrane in Bio-Rad blot apparatus. After blocking with 5% non-fat milk in phosphate-buffered saline-Tween-20 for 1 h at room temperature, the membranes were blotted with the appropriate TGFBR1 ,SMAD7, b-Actin primary antibody (Santa Cruz Biotechnology, Santa Cruz, CA) at a 1:1000–1:2000 dilution. Membranes were then incubated with the appropriate secondary antibody linked to horseradish peroxidase at a 1:2000 dilution for 1 h at room temperature. After TBSTwashes, the blot was incubated in detection reagent (ECL Advance Western Blotting Detection Kit, Amersham Bioscience, Freiburg, Germany) and exposed to a Hyperfilm ECL film (Pierce, Rockford, IL).
